# Common Species Maintain a Large Root Radial Extent and a Stable Resource Use Status in Soil-Limited Environments: A Case Study in Subtropical China

**DOI:** 10.3389/fpls.2020.01260

**Published:** 2020-08-21

**Authors:** Xingyu Ma, Hongsong Chen, Yunpeng Nie

**Affiliations:** ^1^Key Laboratory of Agro-ecological Processes in Subtropical Region, Institute of Subtropical Agriculture, Chinese Academy of Sciences, Changsha, China; ^2^Huanjiang Observation and Research Station for Karst Ecosystems, Chinese Academy of Sciences, Huanjiang, China; ^3^University of Chinese Academy of Sciences, Beijing, China

**Keywords:** southwest China, common species, shallow rocky soil, rooting characteristics, plant adaptation, karst ecosystems

## Abstract

Coarse root systems provide a framework for water and nutrient absorption from the soil and play an important role in plant survival in harsh environments. However, the adaptions of plant roots in soil-limited environments with low water storage capacity and nutrient content needs to be better understood. The adaptation strategies of two common plant species (a deciduous tree *Platycarya longipes* and an evergreen shrub *Tirpitzia ovoidea*) from two contrasting habitats (a shallow rocky soil and a nearby deep soil) in a karst region of subtropical China were compared and analyzed. Foliar nutrient concentrations, stoichiometry, stable carbon, and oxygen isotopes were used to determine plant nutrient and water use status across these two habitats. Six indexes, including maximum root depth, maximum root radial extent, number of major roots and secondary roots, and tapering rate and curvature, were all investigated to characterize coarse root systems. Results show that both species exhibited similar nutrient and water use status in the two habitats that had contrasting water holding capacity and available nutrient content. On the other hand, although maximum root depths of the individual plants were not deeper than 33 cm, maximum radial extents were much larger when compared to rooting depths. Specifically, the ratio of radial extent to depth in the soil-limited habitat was approximately 6 and 1.5 times higher than that in the deep-soil habitat for the tree and shrub, respectively. Additionally, especially for the tree, a larger root radial extent was further accompanied by lower root tapering rate and bending levels. Our results provided evidence that plants growing in soil-limited environments maintain a stable resource use status along with large radially extended coarse root systems in humid karst regions like southwest China.

## Introduction

The growth and survival of any plant is dependent on both above- and below- ground resources (Matyssek et al., 2012). When aboveground resources, such as light and heat, are relatively abundant, the availability of belowground water and nutrients is more critical. Compared with the aboveground of plant (i.e., stems, branches, leaves), roots are much less known because of the difficult, labor-intensive and costly work (Maeght et al., 2013). One of the most important aspects of root systems is the rooting depth. It has long been assumed that the rooting depths of natural vegetation maximize evapotranspiration and are optimized to local climates and soil textures (Milly, 1994). Along with these predicted patterns, based on meta-analysis of global data, it was found that, in wet regions, root systems are shallow compared to infiltration depth; while, in dry regions, root systems are normally deeper and approach maximal infiltration depth (Schenk and Jackson, 2002; Schenk, 2008b). The convergence of theory and fact was investigated for root systems that expand freely in unlimited deep soils (Schwinning, 2010); landforms characterized by shallow soils underlain by hard substrates were usually excluded from consideration.

It is known that plant roots in shallow soil environments absorb water by growing through fractured rock into deeper layers (e.g., Phillips, 1963; Zwieniecki and Newton, 1995; Sternberg et al., 1996; Graham et al., 1997; Hubbert et al., 2001; Graham et al., 2010). With the prevalence of Critical Zone science, plant ecophysiologists have focused on deeper subsurface non-soil resources and the role of deep roots in soil limited environments [recently reviewed by Dawson et al. (2020)]. Karst regions are one of the most typical shallow soil profiles and occupy 10–15% of the total continental area (Ford and Williams, 2013). Most weathered materials that are derived from the soluble carbonated bedrock are removed by water flow, thus resulting in shallow soil coverage (Cao et al., 2005). A series of studies conducted in karst regions provide evidence for the dominance of deep root systems that access groundwater or perched water tables (Jackson et al., 1999; Querejeta et al., 2007; Nie et al., 2011; Estrada-Medina et al., 2013; Gu et al., 2015; Ding et al., 2018). Poot and Lambers (2008), in southwestern Australia, further reported that seedlings of shallow-soil endemics employed specialized root strategies for exploration of deeper resources. Accordingly, deep root penetration is the main strategy employed by plants in soil-limited karst environments.

Not all studies support the viewpoint that deep root penetration is a necessary strategy for plants adapted to shallow soil environments. For example, recent studies suggest that roots of adapted species in typical karst terrains were restricted to shallow soil layers (Heilman et al., 2009; Kukowski et al., 2013; Ni et al., 2015; Dammeyer et al., 2016; Du et al., 2019). Nie et al. (2014a) excavated the coarse root systems of two common species in a karst region of southwest China, and showed that common species, from a variety of habitats, exhibited lateral (almost horizontal) rather than vertical root systems. Furthermore, not all habitats provide fractured substrate. So, unlike endemic species adapted to shallow soils underlain by fractured bedrock, common species, adapting to other types of karst habitats, have developed other rooting characteristics. A complex structure of fine roots is generally considered an indicator of resource absorption and utilization (McCormack et al., 2015). However, in soil-limited environments, where fine roots are difficult to sample at an individual level, investigating and understanding the coarse root pattern is of great significance for understanding plant water and nutrient acquisition (Schenk and Jackson, 2002; Schwinning, 2010; Nie et al., 2011; Maeght et al., 2013). Furthermore, in those soil-limited environments that have a low nutrient content per soil volume, coarse root systems play an even greater role by extending the range of resources acquisition.

The extent of a root system is often predicted by the size of aboveground and climatic regimes, especially by the growth form of the plant (Schenk and Jackson, 2002). For example, rooting depth and lateral root spread generally increase as we progress from herb, to shrub to tree (Peek et al., 2005). Additionally, the root to canopy ratio also differs, i.e. trees have a higher root extent to depth ratio than shrubs. The close relationship between the plant growth form and root dimensions may be due to the resource demands of the plant (Schenk and Jackson, 2005). For example, trees tend to have the deepest and widest lateral spread roots to supply water and nutrient to their leaf biomass. Shrubs are next, then forbs and grasses (Han et al., 2005; Westoby and Wright, 2006). Locally, plant-rooting depths may vary substantially from general patterns. More research needs to be done on the variations in rooting characteristics among species of different plant growth forms. It is understood that trees can obtain additional resources and maintain a stable use of resources because their roots penetrate deeply into bedrock fractures. We need to determine if plants with shallow lateral root systems can also use resources in a stable manner.

One way to determine if a plant can meet normal growth needs in soil-limited habitats is to look at water and nutrient utilization of these same plants in unrestricted environments. Leaf nutrient content and stoichiometry are important indicators of plant nutrient utilization (Koerselman and Meuleman, 1996; Güsewell, 2004). Researches have shown that some species growing in harsh environments have a higher capacity to store N and P than those in moderate environments (Chapin et al., 1990) or they have higher nutrient resorption (Aerts and Chapin, 1999). In other words, once the root systems of a species growing in a soil-limited environment can no longer extract similar levels of nutrients as in a deep-soil environment, foliar nutrient content and/or stoichiometry will change (Henry et al., 2005). In terms of water utilization, studies have suggested that, even for the same species, water use varies based on the type of water environment. The most common regulation method is water use efficiency (WUE), plants tend to enhance WUE under drought stress (Querejeta et al., 2006). Leaf δ^13^C is a good proxy indicator of leaf-level intrinsic WUE, generally, C_3_ plants with high leaf δ^13^C are thought to have high WUE (Dawson et al., 2002). Moreover, a dual isotopic measurement combining δ^13^C and δ^18^O (which is independent of variations in net photosynthetic rate) can help untangle the separate effects of carbon assimilation and stomatal conductance on leaf δ^13^C and WUE and allows for a more comprehensive reflection of plant WUE (Prieto et al., 2018).

Plants adaptation strategies in soil-limited environments was performed on two common species (a deciduous tree, *Platycarya longipes*, and an evergreen shrub, *Tirpitzia ovoidea*) from a shallow rocky soil and a nearby deep soil in a karst region of subtropical China. The main objectives of this study are: (a) to explore whether the common species growing in soil-limited habitats maintain comparable resource use status to the same species growing in nearby deep soils, (b) to reveal the associated characteristics of coarse root systems and the probable differences between habitats, and (c) to investigate whether the environmental effects on two common species are species-specific.

## Materials and Methods

### Study Site

The study was conducted in a small catchment with an area of 1.14 km^2^ in the Huanjiang Observation and Research Station for Karst Ecosystems (24°43’48.8”–24°44’58.9” N, 108°18’58.4”–108°19’56.9” E) administrated by the Chinese Academy of Sciences, located in Guangxi Zhuang Autonomous Region, southwest China. The region experiences a typical subtropical monsoon climate, with a mean annual precipitation of 1389 mm and a mean annual temperature of 19°C. Rainfall mostly occurs from late April to the end of September, which accounts for 74% of the total annual rainfall. This catchment is characterized by a flat depression (approximately 0.06 km^2^) surrounded by mountain ranges, except on the northeast side (the mouth of the catchment). Elevation ranges from 272.0 to 647.2 m. Hillslopes are steep (62% are greater than 25°) and 60% of the slopes are dominated by shallow soil (10–30 cm on average, underlain by weathered or consolidated bedrock) and loose rocky soil (usually a thin layer of coarse gravel underlain by a thick layer of soil and rock fragments) habitats. Rocky outcrops prohibit further expansion of these soil habitats.

The study site was under cultivation before being abandoned at the end of the 1980s. It then experienced approximately 35 years of natural recovery (Chen et al., 2011). Presently, 70% of the hillslopes are dominated by tussocks and shrubs (Nie et al., 2012), and the vegetation shows three secondary communities: tussock, shrub, and secondary forest from uphill to middle and to foot of the slope; there are also some trees sparsely distribute in the middle of the slope, such as *P. longipes* and *Celtis biondii*.

### Habitats and Species Selection

We selected a shallow rocky soil habitat with high rock fragment content (Habitat I) and a nearby deep soil habitat (Habitat II) as comparison. Both of these habitats are located mid-slope and have the same aspect, which excludes the effects of slope position and aspect on plant rooting characteristics. They represent typical habitats of subtropical karst in China. Soil in habitat I is barren and has relatively poor water capacity, while in habitat II, the soil is thicker and has a higher water capacity and resource availability with a more uniformly moderate fertility (**Table 2**).

In habitat I, the plant community is shrubland dotted with a few trees, the main species are *P. longipes*, *T. ovoidea*, *C. biondii*, and *Leptodermis ovata*. While in habitat II, the plant community is mainly composed of trees, the main species were *P. longpies*, *T. ovoidea*, and *Mallotus philippensis*. Based on field investigation, we found that a deciduous tree, *P. longipes*, and an evergreen shrub, *T. ovoidea*, are common species growing in both selected habitats. They are also found to be widely spread in the karst regions of southwest China. Plots (20 m × 20 m) were established for each of the two habitat types, with a distance between plots was 800 m (see **Supplementary Figure S1** for details). Three to six mature individuals (indicated by their ability to set seed) per species per habitat were sampled randomly for further investigation. Basal information of the selected plants is shown in **Table 1**. Specifically, individuals of *P. longipes* in habitat I usually have basal branches, only the DBH of the biggest branch was shown in **Table 1**.

**Table 1:** Major information of the common, dominant species: *P. longipes* and *T. ovoidea*, in two distinct habitats in a karst ecosystem of southwest China.

Species	Family	Habitat	Leaf phenology	Number of basal branches	DBH^*^ (cm)	Height (m)	Estimated age (a)
*P. longipes*	*Juglandaceae*	Habitat I^**^	Deciduous tree	2.5 ± 1.43	6.97 ± 1.63^b^	3.17 ± 0.45^b^	11.33 ± 0.58
		Habitat II		- -	11.95 ± 3.55^a^	5.93 ± 1.11^a^	12.67 ± 2.52
*T. ovoidea*	*Linaceae*	Habitat I	Evergreen shrub	4.3 ± 1.47	2.71 ± 1.03	1.56 ± 0.18^b^	- -
		Habitat II		2.2 ± 1.09	3.23 ± 0.91	2.90 ± 1.15^a^	- -

^*^For tree species, this refers to the diameter at breast height; for shrub species, this refers to the base diameter. Additionally, plants in habitat Ⅰ usually have 2 or 3 basal branches, of which only the largest DBH in these branches were recorded and analyzed, and the stand basal areas were calculated based on all branches of trees in habitat Ⅰ. ^**^Ⅰ, shallow rocky soil (frequently, <30 cm depth), weakly weathered bedrock layer underlain. Ⅱ, nearby deep soil (>50 cm depth), deep and homogenous soil. “- -” means no annual ring data. Means in a column of same species followed by different letters are significantly different according to one-way ANOVA and LSD (p < 0.05). Values are mean ± SD.

### Field Sampling and Laboratory Analysis

In August 2017, mature, sunlit leaves were collected from each sample plant individual of each species in both habitats. There were nine samples (six for *P. longipes* and three for *T. ovoidea*) in habitat I and seven samples (four for *P. longipes* and three for *T. ovoidea*) in habitat II, respectively, for a total of 16 leaf samples. All samples were oven-dried at 70°C to constant weight and ground for further analysis.

Soil samples were also collected in August 2017. Three soil cores (15 cm depth) were taken randomly around each sample root and thoroughly mixed into one composite sample. The composite samples were placed in polyethylene bags and transported to the laboratory. After they were air dried, the soil samples were ground and passed through a 2 mm mesh sieve for physicochemical analysis.

Soil organic carbon (SOC) was measured by the potassium dichromate method. Total soil N (TN) was measured by the Kjeldahl determination, and total soil P (TP) was determined by acid digestion with a H_2_SO_4_+HClO_4_ solution. Alkali-hydrolyzable N (AN) was measured by titration with a dilute solution of H_2_SO_4_ after samples had been extracted with a mixture of FeSO_4_ and NaOH. After samples were extracted with 0.5 M Na_2_CO_3_, available phosphorus (AP) was measured by the molybdenum blue colorimetric method. Concentrations were expressed on the basis of oven-dry soil weight. Gravimetric soil moisture content was determined by drying soil samples in an oven at 105°C for at least 72 h. Leaf N concentration was measured with a flow injection analyzer (FIAstar 5000, FOSS, Sweden). Leaf P concentration was measured with the molybdate/ascorbic acid method. Leaf δ^13^C and δ^18^O were measured by Isoprime isotope ratio mass spectrometer (IRMS; MAT 253, Thermo Fisher Scientific, Inc., GER), in the Key Laboratory of Agro-ecological Processes in Subtropical Region, Institute of Subtropical Agriculture, Chinese Academy of Sciences.

The soil water capacity for the two habitats were cited from the doctoral thesis completed by Fu (2017), which used the grid (80 m × 80 m) sampling method in the studied catchment. In his study, a soil core was used to collect soil samples at five points from the surface (0–15 cm) and around each grid intersection. Parameters such as rock fragment content (RC), soil texture (sand and clay content), and soil organic carbonate (SOC) were measured. It is worth noting that gravel particle sizes that were larger than the diameter of the soil core (38 mm) were generally ignored during sampling. We selected three sample sites around the shallow rocky soil habitat and the nearby deep soil habitat from Fu’s research and then analyzed their mean values of RC, sand and clay content, and SOC to represent the soil physicochemical properties in the two habitats. *Rawls* model (Rawls et al., 1982) was used for estimating the field capacity and wilting coefficient of soil (0–15 cm) in the sample habitats to reduce laboratory work, and after that, available water capacity (AWC) was calculated accordingly. Multiple regression formulas of *Rawls* model are as follows:

(1) $$\theta_{f}^{*}=0.2576-0.0020P_{s}+0.0036P_{c}+0.0299P_{oc}$$

(2) $${\text{θ}}_{w}=0.0260+0.0050P_{c}+0.0158P_{oc}$$

* θ_f_, soil field water capacity. P_s_, soil sand content. P_c_, soil clay content. P_oc_, soil organic carbon. θ_w_, soil wilting coefficient.

### Root Investigation

Root characteristics were investigated by manually excavating intact root systems of the selected plants between August and October 2017. All main roots arising from the base of the target plants were identified first, lateral roots were then exposed by following the exposed main roots. Roots from other plants were easily distinguished as they were not connected to the main root. Roots with diameters of <0.5 cm were not measured because they break easily, and it was difficult to determine their origin. Broken roots were reconnected with adhesive tape. We analyzed the fine root biomass in different soil layers at the community level. However, because of the difficulties in distinguishing which plant the roots emanate from using soil cores and minirhizotron, the fine roots at the individual level were not taken into account in this study.

**Figure 1:**
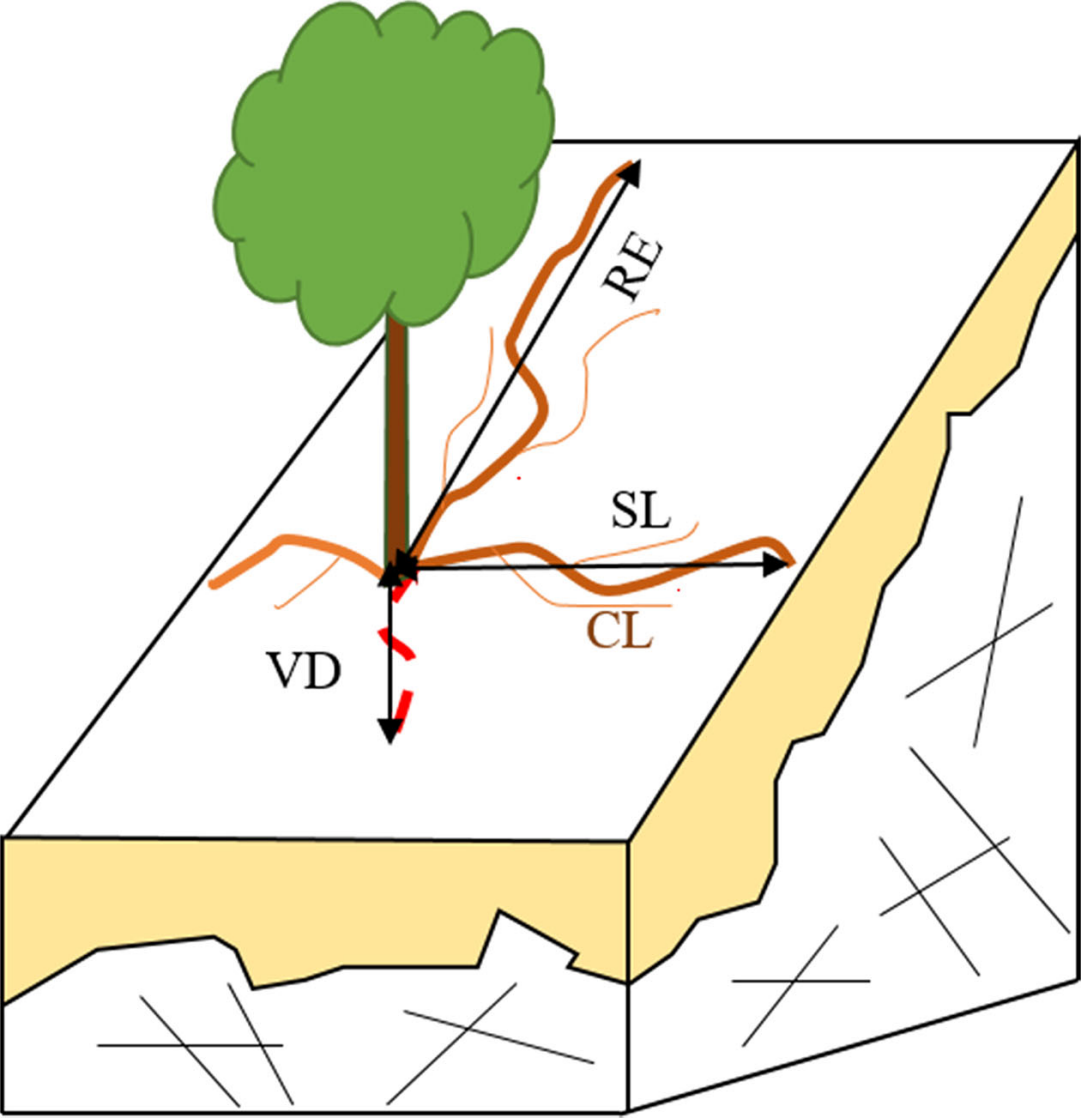
A schematic of parameters in root system investigation. RE, SL, RL, and VD refer to maximum root extend, straight length, curve length and maximum vertical depth, respectively.

When the whole root system was exposed, maximum rooting vertical depth (VD) and maximum radial extent (RE) were both measured. Other parameters, such as curve length (CL), straight length (SL), root diameter (RD), and the number of main and lateral roots, were also recorded. Information on the root investigation indexes is found in **Figure 1**. We measured CL, the length from the base of stem to the distal along the main roots. Roots were then marked at regular intervals according to the CL (every 10 cm for the first 50 cm and every 50 cm after that), and RD was measured based on these marks.

Only mature trees were sampled. The height and diameter of breast height (DBH) varied markedly, which made direct inter-plant comparisons of root structure characteristics impossible. In order to eliminate the impact of the variation in size and compare root structure characteristics, we used two plant size-independent parameters, root tapering pattern, and root curvature (Nie et al., 2014a). Tapering rate and curvature could be calculated as:

(3) $$\text{Tapering rate}=\frac{RD_{n}-RD_{n+1}}{CL_{n+1}-CL_{n}}$$

(4) $$\text{Curvature}=\frac{SL}{CL}$$

RD_x_, RD at the mark X previously made on the root; CL_x_, CL from plant base to the mark X; CL, curve length; SL, straight length.

Root tapering rate reflects the rate at which the root diameter gradually decreases along RL. The root tapering rate ranges from 0 to 1. If the value is close to 0, then the root diameter decreases slowly per length unit. If it is closer to 1, then root diameter decreases sharply. Root curvature can reflect the degree of root bending. A curvature also ranges from 0 to 1, a curvature close to 0 indicates a high root bending degree. While, a curvature closer to 1 indicates a relatively linear root pattern.

Both indices were calculated based on the individual plant features, and then, the average of individuals within a species within one habitat was used for statistical analysis and graphing.

### Statistical Analysis

We tested the impact of soil physical-chemical properties on foliar C, N and P concentrations, stoichiometry characteristics and isotopic natural abundance (δ^13^C and δ^18^O) with a one-way ANOVA followed by the LSD multiple comparison test using at least three replicates per species in each habitat. We calculated the average tapering rate per root segment along CL (separated based on marks on each taproot) and root curvature of each individual. The differences in tapering rate and curvature for each species in different habitats were compared based on the fact that these two parameters showed no significant difference for the same species in the same habitat. Statistical analyses were performed using SPSS 19.0 (SPSS Inc., Chicago, IL, USA) and Origin 9.0 (OriginLab, Hampton, MA, USA).

To evaluate the key factors of plant adaptation strategy in soil-limited habitats, we performed a redundancy analysis (RDA) and extracted the scores on the first and second axes of variation of the RDA and the contribution of every trait related to plant adaptive strategy. The statistical significance of RDA results was determined using the Monte Carlo permutation method based on 999 runs with randomized data. Results were considered as statistically significant if *p* < 0.05.

## Results

### Soil Moisture and Nutrient Conditions in Two Habitats

As shown in **Table 2**, significantly shallower soil depth and lower AWC were found in habitat I than in habitat II. Additionally, soil SOC, TP and AP in habitat II were around 3 times higher than in habitat I, and habitat II also exhibited significantly higher soil TN and AN than that in habitat I. Moreover, contrasted to the nearby soil habitat with extremely low gravel content, the gravel content in habitat I reached about 50% (data not shown). Additionally, ignoring gravel with a diameter greater than 38 mm led to a greater difference in water and nutrient conditions between the two habitats than was recorded in **Table 2**.

**Table 2:** Comparison of soil water holding capacity, moisture conditions and soil nutrient concentrations in two selected habitats in a southwest China karst ecosystem.

Soil condition parameters	Habitat Ⅰ^*^	Habitat Ⅱ
**Soil depth (SD)(m)**	0.32 ± 0.04^b^	0.70 ± 0.11^a^
**Bulk density (BD) (g·cm^-3^)**	1.46 ± 0.02^a^	0.68 ± 0.01^b^
**Rock fragment content (RC) (%)**	30.83 ± 6.57^a^	10.36 ± 6.47^b^
Filed water capacity ^**^ (θ_f_) (%)	27.31 ± 0.25^b^	34.32 ± 0.48^a^
Wilting coefficient ^**^ (θ_w_) (%)	14.83 ± 0.13^a^	18.06 ± 0.28^a^
Available water capacity ^**^ (AWC) %	12.48 ± 0.03^b^	16.26 ± 0.03^a^
**SOC (g.kg^-1^)**	24.74 ± 0.09^b^	78.04 ± 16.06^a^
**TN (g.kg^-1^)**	0.31 ± 0.15^b^	0.62 ± 0.12^a^
**TP (g.kg^-1^)**	0.25 ± 0.08^b^	0.88 ± 0.06^a^
**AN (mg.kg^-1^)**	333.42 ± 92.76^b^	766.09 ± 92.29^a^
**AP (mg.kg^-1^)**	3.40 ± 0.67^b^	9.78 ± 0.48^a^

*Ⅰ, shallow rocky soil habitat; Ⅱ, nearby normal deep soil habitat. ** Results of the Rawls model. SOC, soil organic carbon; TP, total soil phosphorus; TN, total soil nitrogen; AN, available nitrogen; AP, available phosphorus; AK, available potassium. Means in a line followed by different letters are significantly different according to one-way ANOVA and LSD (p < 0.05). For AN and AP, n=4 in both habitat Ⅰ and habitat Ⅱ, for other 8 soil condition parameters, n=9 and 7 for habitat Ⅰ and habitat Ⅱ, respectively. Values are mean ± SD.

### Leaf Chemical Characteristics in Two Habitats

As shown in **Table 3**, there were no significant differences in foliar C, N, P for the same species in different habitats, or in values of C/N, C/P, N/P. The foliar N:P ratio was significantly lower in *P. longipes* than in *T. ovoidea*, while both of them were <14, reflecting a N limitation for both of the species in both habitats.

**Table 3:** Foliar nutrient concentrations and stoichiometric characteristics of two habitats in southwest China karst ecosystem.

Stoichiometry ^*^	*P. longipes*		*T. ovoidea*	
	Habitat Ⅰ	Habitat Ⅱ	Habitat Ⅰ	Habitat Ⅱ
Foliar C (g·kg^-1^)	46.50 ± 2.18	48.41 ± 0.63	46.35 ± 0.01	46.33 ± 0.05
Foliar N (g·kg^-1^)	1.88 ± 0.09	1.70 ± 0.17	2.44 ± 0.19	2.50 ± 0.18
Foliar P (g·kg^-1^)	0.20 ± 0.02	0.22 ± 0.02	0.22 ± 0.01	0.21 ± 0.01
C/N	24.83 ± 1.76^a^	28.66 ± 2.99^a^	19.04 ± 1.45^b^	18.55 ± 1.31^b^
C/P	232.77 ± 29.92	225.69 ± 16.88	212.45 ± 5.54	222.80 ± 0.55
N/P	9.36 ± 0.85^b^	7.93 ± 0.96^b^	11.20 ± 1.14^a^	12.04 ± 0.88^a^

*Means in a line followed by different letters are significantly different according to one-way ANOVA and LSD (p < 0.05). Values are mean ± SD.

**Figure 2:**
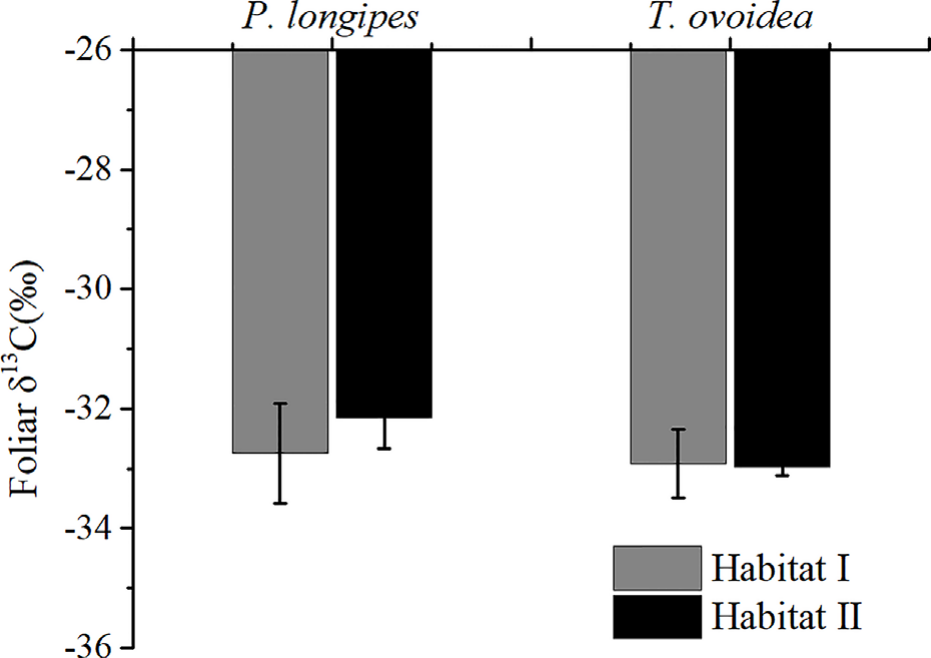
Comparison of mean foliar δ^13^C values for *P. longipes* and *T. ovoidea* growing in the two selected habitats. “Habitat I” indicates values obtained in shallow rocky soil habitat, whereas “Habitat II” refers to values obtained in the nearby deep soil habitat. *Error bars* represent ± 1 SD (n = 9 and 7 for habitat I and habitat II, respectively). There is no significant difference between species or habitats (*p* < 0.05).

**Figure 3:**
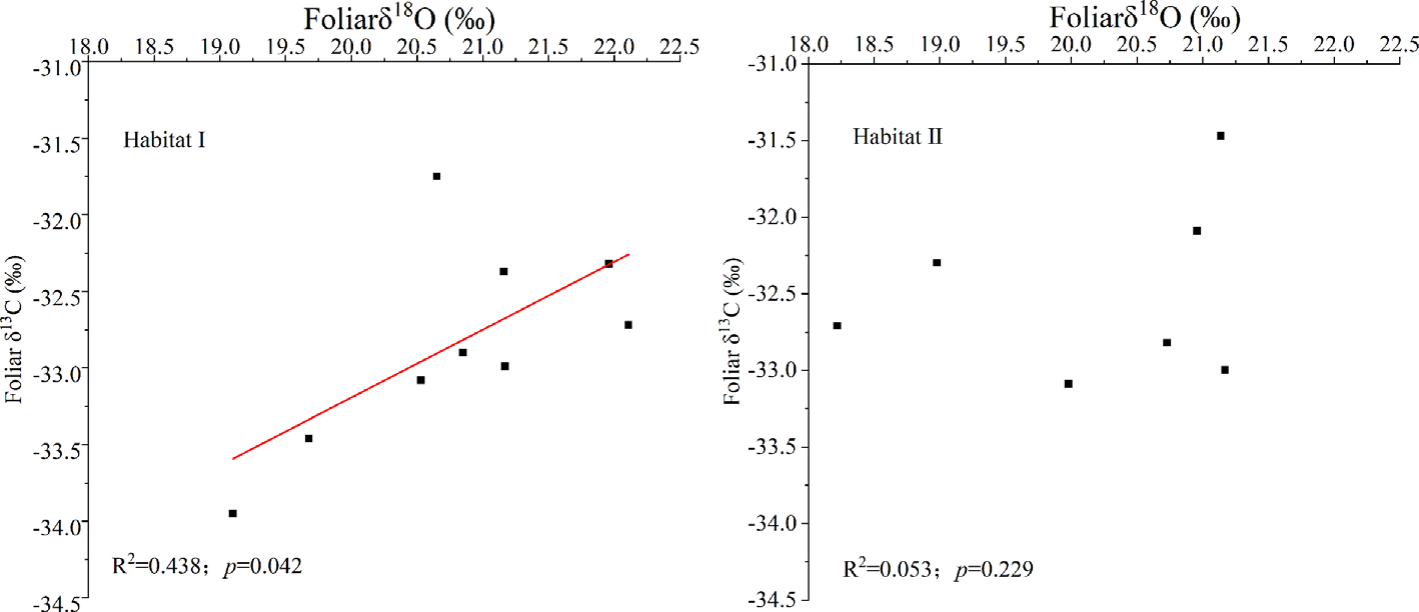
Linear regression of the foliar δ^13^C and δ^18^O values for all of the studied plants (all dots) in habitat I and habitat II. “Habitat I” refers to values obtained in shallow rocky soil habitat, and “Habitat II” refers to values obtained in the nearby deep soil habitat.

**Figure 2** showed that species growing in habitat I exhibited similar foliar δ^13^C values as those growing in habitat II. Specifically, around -32.44 ± 0.67‰ for *P. longpies* and -32.94 ± 0.36‰ for *T. ovoidea*. Moreover, leaf δ^13^C was positively correlated with leaf δ^18^O across species in habitat I while not in habitat II (**Figure 3**).

### Root Architectural Traits

Both of the common species growing in the selected karst habitats exhibited relatively shallow root systems as VD of all the individuals were less than 33 cm (**Table 4**). Additionally, the average RE of the two habitats were about 9 and 1.5 times greater than VD for *P. longipes* and *T. ovoidea*, respectively.

**Table 4:** Coarse root parameters for two common species in two selected habitats in southwest China.

Root Parameters	*P. longipes*		*T. ovoidea*	
	Habitat Ⅰ	Habitat Ⅱ	Habitat Ⅰ	Habitat Ⅱ
Number of major roots (NMR)	5.16 ± 1.53^a^	3.50 ± 1.00^b^	4.33 ± 1.16a^b^	5.67 ± 0.58^a^
Number of secondary roots (NSR)	4.22 ± 0.31^b^	6.29 ± 0.60^a^	0.69 ± 0.10^d^	1.00 ± 0.01^c^
Maximum root radial extent (RE) (m)	3.12 ± 1.06^a^	1.07 ± 0.17^b^	0.33 ± 0.19^c^	0.32 ± 0.08^c^
Maximum vertical depth (VD) (m)	0.22 ± 0.07	0.24 ± 0.08	0.20 ± 0.02	0.23 ± 0.03
Ratio of root radial extent to vertical depth (RE/VD)^*^	15.65 ± 7.44^a^	2.94 ± 1.18^b^	1.65 ± 0.34^c^	1.07 ± 0.04^d^
Canopy width (CW) (m)	2.1 ± 0.44^b^	2.9 ± 0.74^a^	0.53 ± 0.06^c^	1.53 ± 0.06^b^
Ratio of root radial extent to canopy width (RE/CW)^**^	1.50 ± 0.52^a^	0.61 ± 0.32^b^	0.61 ± 0.10^b^	0.21 ± 0.11^c^

*Results of maximum root radial extent versus maximum vertical depth; **results of maximum root radial extent versus canopy. Means in a line followed by different letters are significantly different according to one-way ANOVA and LSD (p < 0.05). Values are mean ± SD.

On the other hand, the lateral root range of both of the species in habitat I were wider than that in habitat II. Especially for *P. longipes*, RE in habitat I was almost three times greater than that in habitat II. Additionally, this extensive radial root extent in habitat I was accompanied by several other parameters. The ratios of root radial extent to depth in habitat I were approximately 6 and 1.5 times greater than that in habitat II for *P. longipes* and *T. ovoidea*, respectively. And, the ratios of root radial extent to canopy width in habitat I were 2.5 and 2.9 times higher than that in habitat II for *P. longipes* and *T. ovoidea*, respectively. Even though the height of plants growing in habitat II were significantly lower (**Table 1**), they had significantly larger root radial extension range than those in habitat II.

**Figure 4:**
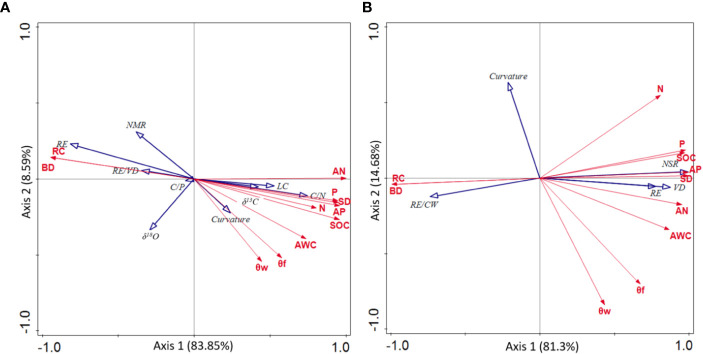
Redundancy analysis (RDA) of leaf nutrient status, water use efficiency, and root structure parameters for adaptation strategy of *P. longipes* **(A)** and *T. ovoidea* **(B)** in two contrasting habitats. Depicted are the soil environmental variables (red arrows) and plant adaptation trait variables (blue arrows). Abbreviations for environmental variables are given in, and abbreviations for leaf nutrient status and root parameters are shown in and.

The correlations between plant adaptation traits and environmental factors were shown in **Figure 4**. Plant adaptation traits, which were correlated to environmental factors, were almost always related to root patterns, which was consistent to the findings shown in **Table 4**. Specifically, the main environmental factors affecting plant adaptability were RC, bulk density (BD), AN and soil depth (SD). In addition, RE (*P* = 0.01, *F* = 10.3) and RE/CW (*P* = 0.11, *F* = 3.2) were the strongest quantitative indicators in support of the adaptation of *P. longipes* and *T. ovoidea* to soil-limited habitats (with high RC and BD, low AN and SD), respectively.

### Root Mechanical Traits

**Figure 5:**
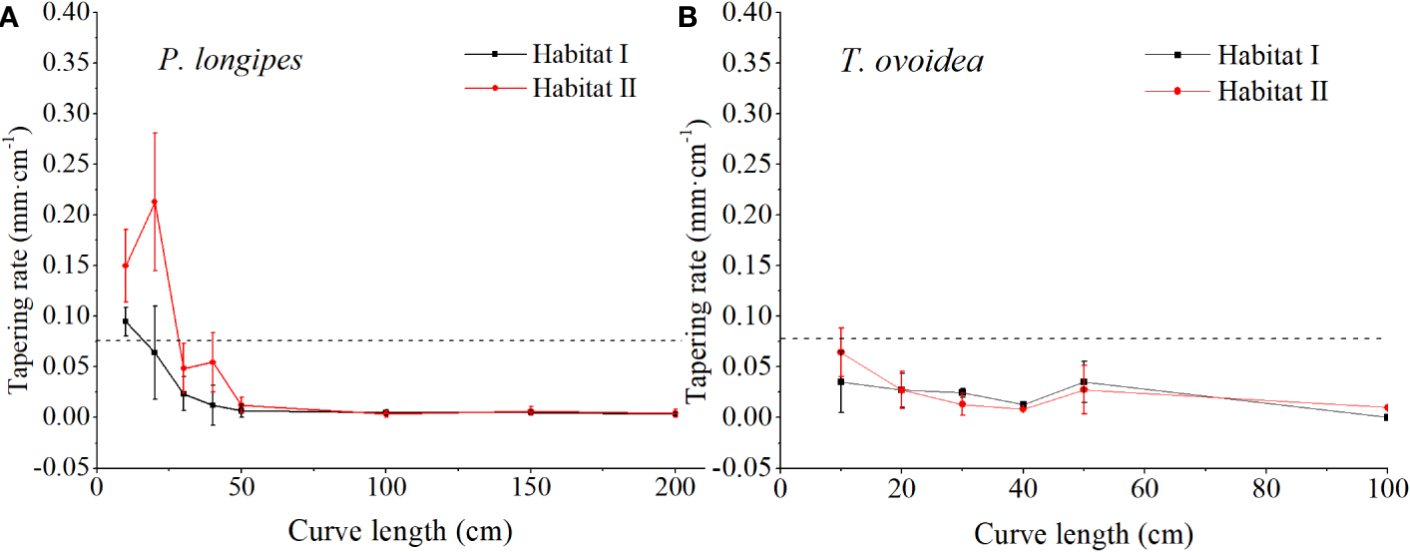
Continuous fluctuation of root tapering rate with increasing root curve length for *P. longipes* **(A)** and *T. ovoidea* **(B)** growing in the two selected habitats. *Error bars* represent ± 1 SD. *Dotted lines* parallel to the axes of the curve length separate root tapering rates greater than or less than 0.075 mm cm^-1^.

As shown in **Figure 5**, in the 0–40 cm section of CL, tapering rates at each root section for *P. longipes* growing in habitat I were significantly lower than that in habitat II. Specifically, in habitat I, all sections of CL (0–200 cm) were plotted below 0.075 mm cm^-1^ except the 0–10 cm section, which favored a horizontal extension of roots in the shallow rocky soil habitat. While in habitat II, although the root tapering rates in the section of 50–200 cm were similar to that in habitat I, they were higher within the section of 0–40 cm (the largest rate was 0.351 at 20 cm, and the average rates were ranging from 0.048 to 0.213), which matched their relatively small lateral extension range.

Contrary to the large variation for *P. longipes* growing in different habitats, root tapering patterns of *T. ovoidea* exhibited flat patterns. More specifically, roots of *T. ovoidea* growing in both habitats tapered slightly and barely fluctuated, all sections within the total CL of 0–100 cm were plotted below the line at 0.075 mm cm^-1^.

**Figure 6:**
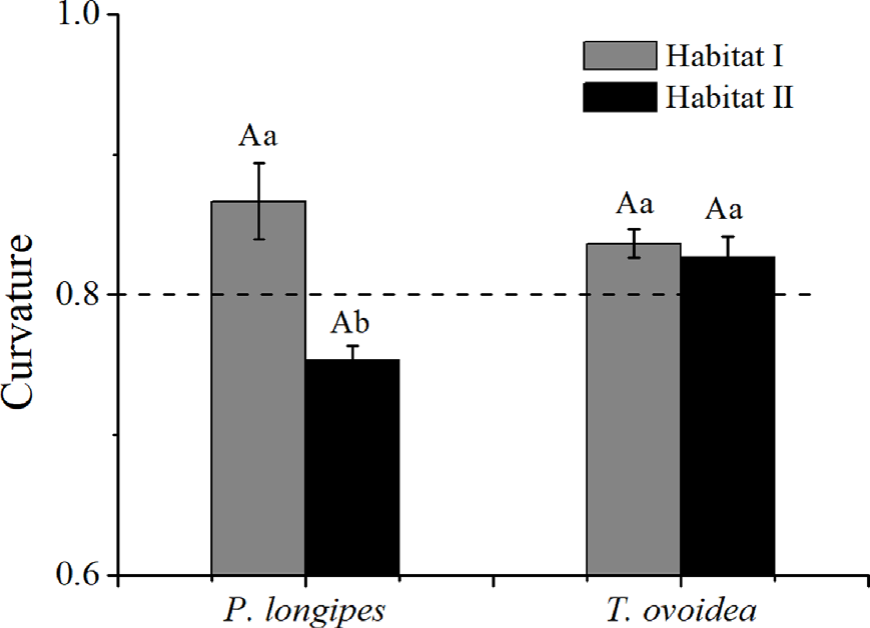
Mean root curvature of *P. longipes* and *T. ovoidea* from the two habitats. *Error bars* represent ± 1 SD (n = 9 and 7 for habitat I and habitat II, respectively). *Dotted lines* parallel to the axes of the species separate curvatures that are greater than or less than 0.8. Lowercase letters in the same species stand for significant differences between different habitats (*p* < 0.05). Capital “A” and lowercase “a” denote statistical significance in two habitats and two species, respectively.

As shown in **Figure 6**, the root curvature of *P. longipes* growing in habitat I (0.87 ± 0.03) was significantly greater than that in habitat II (0.75 ± 0.01), which reflected a slighter root bending degree of *P. longipes* in habitat I than that in habitat II.

Contrary to the obvious differences for *P. longipes*, root curvatures of *T. ovoidea* growing in the two habitats exhibited similar patterns and both of them were higher than 0.8, indicated that the root extension process of *T. ovoidea* in both habitats exhibited an approximately linear form.

## Discussion

In the current study, two common species (the tree *P. longipes* and the shrub *T. ovoidea*) growing in a typical rocky habitat and a nearby deep soil habitat were studied to investigate plant adaptations to environmental stress. We found that there was no significant difference in time-integrated WUE (**Figure 2**) or nutrient status on the leaf level (**Table 3**) in common species growing in the two contrasting habitats. Our results were contrary to some other studies which found notable differences in water use efficiency (Prieto et al., 2018; Querejeta et al., 2018), nutrient resorption (Brant and Chen, 2015) and stoichiometry (Elser et al., 2000) under different environmental conditions. Which leads one to believe that the undifferentiated characteristics of water and nutrient utilization were related to the plant root adaptations.

Both common species were characterized by shallow root systems in both habitats. Additionally, the maximum root depth of both *P. longipes* and *T. ovoidea* in both habitats was less than 33 cm (**Table 4**). Studies have suggested that the both species are widely found in peaks and the upper hillslopes of karst in southwest China (Yu, 2003; Rong et al., 2012; Du et al., 2017; Ding et al., 2018), with extremely high bare rock (Cao et al., 2014). Based on these results, one would speculate that the survival of these two species in a rocky habitat would benefit from deep roots which penetrate into cracks (Yu, 2003), which is contrary to our findings. Other studies reported that plants prefer deep root penetration to compensate for the shallow soil limitation (Jackson et al., 1999; Querejeta et al., 2007; Schwinning, 2013; Gu et al., 2015). While, other researchers agree with our findings, in karst regions, fine and coarse plant roots were distributed in the shallow soil layer (Heilman et al., 2009; Ni et al., 2015; Nie et al., 2017; Du et al., 2019) where the soil is characterized by higher nutrient content, warmer temperature, and roots require less carbon (Steele et al., 1997). These conflicting viewpoints are due to habitat differences and root plasticity of the plant species (Mulia et al., 2010; Isaac et al., 2014). Generally, endemic species in shallow-soil habitat resort to specialized root strategies associated with increasing root biomass close to the bedrock surface to increase their penetration into deeper layers (Poot and Lambers, 2008; Schwinning, 2013). Compared with endemic species, widespread species, like the common species in our study, normally have higher adaptive plasticity (Bastida et al., 2013; Hand et al., 2017), thus, they exhibit varied root characteristics in different habitats (Li et al., 2017).

Compared with the undifferentiated adaptation characteristics of rooting depth, the range of root laterally extent exhibited significantly different traits for each species in the two habitats (**Table 4** and **Figure 5**). Specifically, both species that were growing in soil-limited habitat, have a longer radially extending root than in the nearby deep soil habitat. In the unfavorable conditions for deep rooting, plants need to explore other root characteristics to obtain sufficient soil resources for growth (Semchenko et al., 2018), especially, in a habitat with low water and nutrient contents per soil volume. For example, the shallow rocky soil habitat in our study is characterized by thin soil overlying weakly weathered and shallow bedrock. Previous researches confirmed that when the underlying surface is unfavorable for root penetration, roots are concentrated in the topsoil (Schenk, 2008a; Schenk, 2008b) and favor horizontal extensions (Stone and Kalisz, 1991; Martre et al., 2002). In addition, because of the extremely low water storage capacity per soil volume in the shallow rocky soil habitat, most of the water is lost through subsurface flow occurring in the soil-epikarst interface (Fu et al., 2016; Wang et al., 2019). Even in heavy rainfall events, only small amount of rainfall is stored. Therefore, it is the rainfall frequency, which determines the availability of water to plants in these soil-limited habitats, rather than the rainfall amount. Moreover, given that there are more frequent light rainfall events in this region (Yang et al., 2012), coupled with canopy interception, only shallow soil moisture is replenished. As a consequence, plants obtain a larger range of shallow soil water through the laterally extending root system, not only as an adaptation to the geotechnical environment but also to the rainfall characteristics of the region. Additionally, at least from the aspects of low energy consumption for construction, maintenance, and resource uptake, shallow root systems have an advantage over deep roots (Schenk, 2008b). Furthermore, various studies indicated that wide lateral roots are more efficient for anchoring plants (Ennos, 1993; Schenk and Jackson, 2002).

In addition to the maximum radial root extension, other coarse root parameters, such as the ratio of root extent to canopy width, and root tapering rate and curvature, further facilitate the expansion of horizontal root range in the soil-limited habitat. Specifically, both *P. longipes* and *T. ovoidea* had a significantly greater ratio of root extent to canopy width in the shallow rocky soil habitat than that in the deep soil habitat (**Table 4**). According to optimal partitioning theory, plants allocate more biomass to belowground organs when they are experiencing water and nutrient deficit (Reynolds and Thornley, 1982; Lezberg et al., 1999). Just as the root hydrotropism strategy results from water deficit (Baluška et al., 2009), in our study, the high root extent to canopy width ratio also plays an important role for plant survival in water scarcity environments (Poorter et al., 2012; Ledo et al., 2018). Moreover, the gradually tapering and slightly curving roots of the two species in the shallow rocky soil habitat increase the possibility of encountering water and nutrients (Nie et al., 2014a; O’Donnell et al., 2015). Benefiting from the lateral extension, roots can explore a wider resource area and increase the chances of achieving a similar water and nutrient use status as found in a deep soil habitat. Overall, the results of RDA suggested that both species adapted to the environment by root pattern modifications. Specifically, *P. longipes* and *T. ovoidea* growing in habitats characterized by shallow soil with high rock content and bulk density favor large root extension and high ratio of root extent to canopy width, respectively (**Figure 4** and **Table 4**). Both species growing in the deep soil habitat also exhibit shallow root systems like those growing in the soil-limited habitat, but no large coarse lateral extension roots. They have a greater number of secondary roots and a higher bending degree, which was conducive to using soil resources found around the basal stem efficiently (Pregitzer et al., 2002; Shan et al., 2007; Nie et al., 2014a; Kong et al., 2016; Valverde-Barrantes et al., 2017).

Although, the distinction in root structures of both species in the two contrasting habitats were consistent, the difference in *P. longipes* was more obvious than in *T. ovoidea* (**Table 4**). This discrepancy relates to the plant growth form (tree and shrub), the leaf phenology (deciduous and evergreen), and/or the aboveground plant size (Peek et al., 2005). Generally, plants with bigger aboveground mass require more resources for sustaining growth and respiration than those with smaller aboveground mass (Han et al., 2005). In order to satisfy the greater resource demand, larger aboveground species need to develop a relatively large root system (Westoby and Wright, 2006). Previous studies in karst regions showed that during the early stages of restoration in with infertile soil conditions, plants have not yet developed large root systems, only small shrubs can be supported to survive and thrive (Wen et al., 2015; Zhang et al., 2015), which has also been shown in non-karst studies (A. G. T, 1916; Glenn-Lewin et al., 1993; Jiang et al., 2018). In terms of leaf phenology, compared with the evergreen species, deciduous species require more resources during the rainy season because of their concentrated growth (Hasselquist et al., 2010; Nie et al., 2014b; Wang and Moore, 2014; Delzon, 2015; Salazar-Tortosa et al., 2018). Therefore, *P. longipes* needs to build a wider range of coarse roots than *T. ovoidea* to assist the distal roots search for a wider range of resources, especially in shallow soils that have low availability of with low resources and weak weathered bedrock where roots cannot penetrate (**Table 2**).

Although our results indicated that common plants adapt to soil-limited environments by developing large radial root extent, it still faces some uncertainties due to the lack specific investigation results into fine roots on an individual level. Firstly, in our study, almost all the root on a community level was found in the top 30 cm of the shallow soil layer (**Supplementary Figure S2**), however, there might be a small amount of the fine roots distributed in relatively deep layers. Which might lead us to underestimate the ability of plant to absorb deep storage water (Nie et al., 2011; Ding et al., 2018). Secondly, the horizontal distribution of the fine roots on an individual level were also difficult to ascertain precisely. Generally, the fine roots might prefer to concentrate in the soil patches with higher water and nutrient contents (Robinson, 1994; Novoplansky, 2019), rather than distribute equably to a homogeneous water and nutrient absorption, which is considered as a foraging adaptation of roots (Hutchings and Kroon, 1994). Therefore, our study does not sufficiently demonstrate the resource acquisition and utilization strategy of plants without considering the fine roots and prevents the further understanding of plant adaptation mechanism in soil-limited environments.

## Conclusions

To reveal how plants adapt to environmental stress, especially in soil-limited environments, we selected two common species that grow in a shallow rocky soil habitat and a nearby deep soil habitat, measured their foliar chemical indexes and investigated their root structures. Our results showed that the same species exhibited a similar level of water and nutrient utilization in the two contrasting habitats that had greatly different soil water holding capacity and available nutrient content. They also had a wide lateral root extension and a high root to canopy ratio in the shallow soil layer, rather than deep penetration. Specifically, the wide root horizontal range of the tree in the soil-limited habitat was accompanied by low root tapering rate and bending degree. Moreover, the tree species has a more obvious difference in root dimension between contrasting habitat than the shrub. These results provided an interface to study the role of root structure in adapting to environmental water and nutrient restrictions and are essential for sustainable silviculture and exploring vegetation adaptability. Future studies combined the absorptive distal roots and transportive proximal roots may shed more light on plant adaptation strategy and mechanism in in soil-limited environments.

## Data Availability Statement

The raw data supporting the conclusions of this article will be made available by the authors, without undue reservation.

## Author Contributions

YN and HC contributed conception and design of the study. XM organized the database and performed the statistical analysis. XM wrote the first draft of the manuscript. All authors contributed to the article and approved the submitted version.

## Funding

This research was funded by the National Natural Science Foundation of China (41930866 and 31971438), the Guangxi Natural Science Foundation (2018GXNSFGA281003), International Partnership Program of Chinese Academy of Sciences (132852kysb20170029), and the Youth Innovation Promotion Association (2018397) of the Chinese Academy of Sciences.

## Conflict of Interest

The authors declare that the research was conducted in the absence of any commercial or financial relationships that could be construed as a potential conflict of interest.
