# Supplementary material for: Common Species Maintain a Large Root Radial Extent and a Stable Resource Use Status in Soil-Limited Environments: A Case Study in Subtropical China
Source: Front Plant Sci. 2020 Aug 21;11:1260. doi: 10.3389/fpls.2020.01260 (PMC7472101; doi:10.3389/fpls.2020.01260)
Supplement: Supplementary file 1 [file Image_1.pdf]

Common Species Maintain a Large Root Radial Extent and a Stable Resource Use Status in Soil-limited Environments: A Case Study in Subtropical China

Xing-yu Ma[1,2,3], Hong-song Chen[1,2], Yun-peng Nie[1,2*]

[1]Key Laboratory of Agro-ecological Processes in Subtropical Region, Institute of Subtropical Agriculture, Chinese Academy of Sciences, Changsha, China
[2]Huanjiang Observation and Research Station for Karst Ecosystems, Chinese Academy of Sciences, Huanjiang, Guangxi, China
[3]University of Chinese Academy of Sciences, Beijing, China

*** Correspondence:**
Yun-peng Nie
nyp@isa.ac.cn

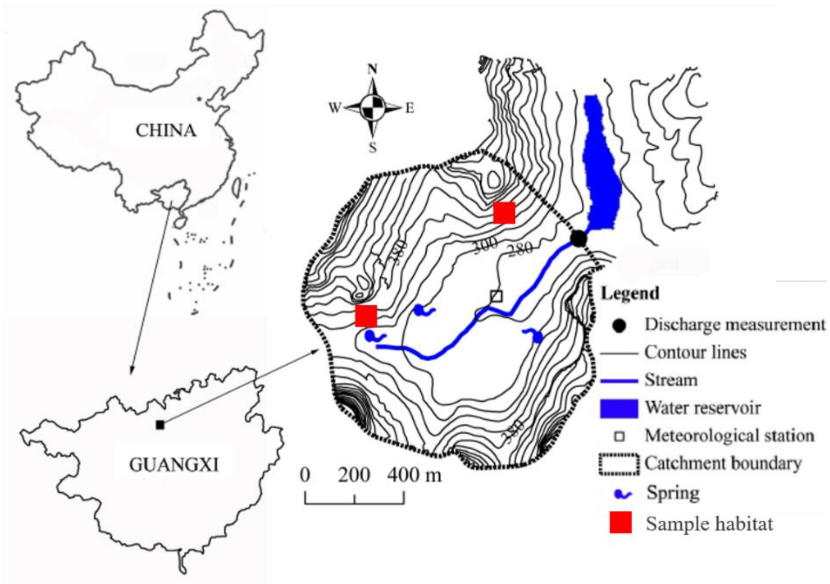

FIGURE S1 | Location of the study area and the plots for each of the two selected habitats (cited and revised from Fu's dissertation, Chinese Academy of Science, 2016). The size of each plot is 400 m^2 (20 m × 20 m)

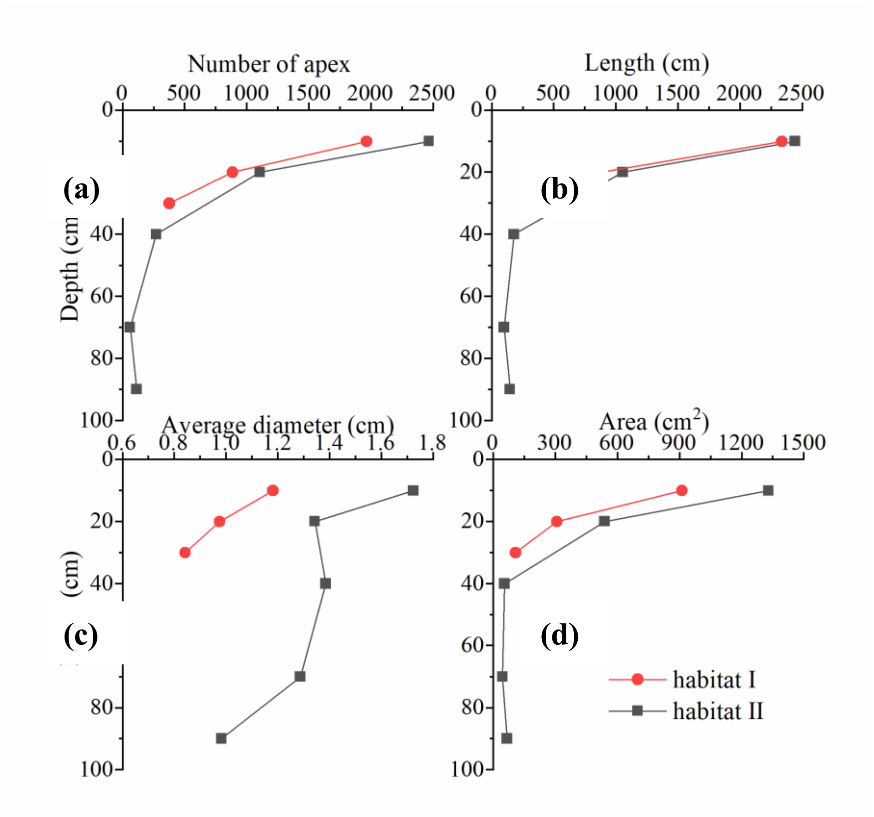

FIGURE S2 | Vertical distribution of fine root parameters in the two community plots nearby habitat I and habitat II. Number of apex (a), length (b), average diameter (c) and area (d). Data on fine root was obtained by soil auger on community level.
